# Factor Analysis of Patients Who Find Tablets or Capsules Difficult to Swallow Due to Their Large Size: Using the Personal Health Record Infrastructure of Electronic Medication Notebooks

**DOI:** 10.2196/54645

**Published:** 2024-04-24

**Authors:** Masaki Asano, Shungo Imai, Yuri Shimizu, Hayato Kizaki, Yukiko Ito, Makoto Tsuchiya, Ryoko Kuriyama, Nao Yoshida, Masanori Shimada, Takanori Sando, Tomo Ishijima, Satoko Hori

**Affiliations:** 1 Division of Drug Informatics Faculty of Pharmacy and Graduate School of Pharmaceutical Sciences Keio University Tokyo Japan; 2 harmo Co, Ltd Tokyo Japan

**Keywords:** tablet, tablets, capsules, capsule, size, personal health record, electronic medication notebook, patient preference, drug, drugs, pharmacy, pharmacies, pharmacology, pharmacotherapy, pharmaceutic, pharmaceutics, pharmaceuticals, pharmaceutical, medication, medications, preference, preferences, pill, pills, machine learning, decision tree, swallow, swallowing, throat, pharynx, risk, risks, dysphagia, speech, mobile phone

## Abstract

**Background:**

Understanding patient preference regarding taking tablet or capsule formulations plays a pivotal role in treatment efficacy and adherence. Therefore, these preferences should be taken into account when designing formulations and prescriptions.

**Objective:**

This study investigates the factors affecting patient preference in patients who have difficulties swallowing large tablets or capsules and aims to identify appropriate sizes for tablets and capsules.

**Methods:**

A robust data set was developed based on a questionnaire survey conducted from December 1, 2022, to December 7, 2022, using the harmo smartphone app operated by harmo Co, Ltd. The data set included patient input regarding their tablet and capsule preferences, personal health records (including dispensing history), and drug formulation information (available from package inserts). Based on the medication formulation information, 6 indices were set for each of the tablets or capsules that were considered difficult to swallow owing to their large size and concomitant tablets or capsules (used as controls). Receiver operating characteristic (ROC) analysis was used to evaluate the performance of each index. The index demonstrating the highest area under the curve of the ROC was selected as the best index to determine the tablet or capsule size that leads to swallowing difficulties. From the generated ROCs, the point with the highest discriminative performance that maximized the Youden index was identified, and the optimal threshold for each index was calculated. Multivariate logistic regression analysis was performed to identify the risk factors contributing to difficulty in swallowing oversized tablets or capsules. Additionally, decision tree analysis was performed to estimate the combined risk from several factors, using risk factors that were significant in the multivariate logistic regression analysis.

**Results:**

This study analyzed 147 large tablets or capsules and 624 control tablets or capsules. The “long diameter + short diameter + thickness” index (with a 21.5 mm threshold) was identified as the best indicator for causing swallowing difficulties in patients. The multivariate logistic regression analysis (including 132 patients with swallowing difficulties and 1283 patients without) results identified the following contributory risk factors: aged <50 years (odds ratio [OR] 1.59, 95% CI 1.03-2.44), female (OR 2.54, 95% CI 1.70-3.78), dysphagia (OR 3.54, 95% CI 2.22-5.65), and taking large tablets or capsules (OR 9.74, 95% CI 5.19-18.29). The decision tree analysis results suggested an elevated risk of swallowing difficulties for patients with taking large tablets or capsules.

**Conclusions:**

This study identified the most appropriate index and threshold for indicating that a given tablet or capsule size will cause swallowing difficulties, as well as the contributory risk factors. Although some sampling biases (eg, only including smartphone users) may exist, our results can guide the design of patient-friendly formulations and prescriptions, promoting better medication adherence.

## Introduction

Tablets and capsules are widely used portable drugs. However, the size and shape of these preparations can pose challenges for some patients, leading to difficulties in swallowing and, consequently, suboptimal medication adherence [1]. To improve this situation, it is necessary to share patient preferences regarding tablets or capsules related to the difficulty of swallowing tablets or capsules, such as what size of tablet or capsule product is best for patients and which patients are likely to find tablets or capsules difficult to swallow because of their size. Therefore, several studies have been conducted on the difficulty of swallowing tablets and capsules owing to their size [1-5]. For instance, Kabeya et al [4], in a survey based on 40 patients, identified that “long diameter (longer oval diameter) + short diameter (shorter oval diameter) + thickness” as the best index and a size of less than 22 mm and thickness of 2 to 6 mm was easy for patients to swallow. Sugiyama et al [2], using endoscopic evaluation in healthy subjects, showed increased dysphagia in patients prescribed tablets exceeding 7 mm in diameter. However, these previous studies have notable limitations, including small sample size [1-4], limitations in reflecting real-world patient experiences [1-4], difficulties in accurately identifying patient backgrounds and medications taken because the information is collected primarily through questionnaires [2-4], and reporting bias [5].

To overcome these limitations, we focused on “electronic medication notebooks” to directly collect information on patient preferences regarding tablets and capsules. In Japan, the introduction of the “medication notebook” system has revolutionized the way patients and health care providers document medication use, precautions, and side effects [6]. This prevents side effects and duplicate administration of multiple medications. In recent years, “electronic medication notebook,” which are “medication notebook” in electronic form using functions such as smartphones, have become widespread [7]. These electronic medication notebooks effectively serve as personal health records (PHR) and enable centralized management of the contents of dispensed medications. The electronic medication notebook also enables secondary uses, such as analysis of the accumulated PHR as big data. Furthermore, questionnaires can be sent directly to patients using an electronic medication notebook as a medium, and PHR, such as dispensing histories, can be collected simultaneously as the answers to questionnaires are sent out. However, the use of PHR data based on electronic medication notebooks has not been explored yet.

Owing to the advantages of the electronic medication notebook, we hypothesized that using the electronic medication notebook would reflect the patient assessment in the real world and accurately identify patient backgrounds and medications taken.

In this study, we collected patient preferences regarding tablets or capsules related to “tablets or capsules difficult to swallow due to their large size” from patients themselves via questionnaires sent through the electronic medication notebook app. The purpose of this study was to clarify the appropriate size of these pharmaceutical forms and to identify factors contributing to swallowing difficulties in patients who “find tablets or capsules difficult to swallow due to their large size.”

## Methods

### Data Sets and Collection Items

**Figure 1:**
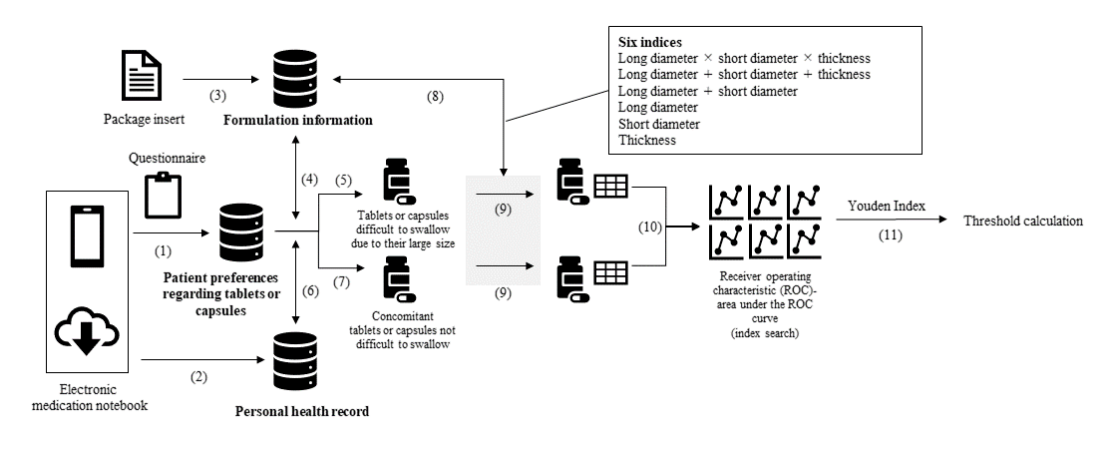
Analysis flow: identification of indices and threshold of the size of tablets or capsules difficult to swallow. ROC: receiver operating characteristic.

**Figure 2:**
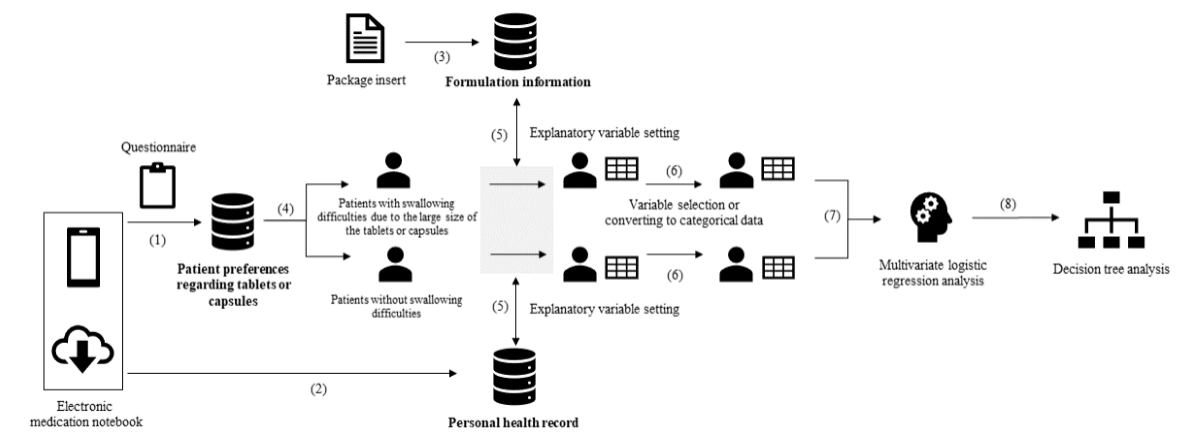
Analysis flow: factor analysis in patients who tend to face swallowing difficulties because of tablet or capsule sizes.

A data set encompassing the needs of the patients regarding tablets or capsules was created through a questionnaire survey conducted from December 1, 2022, to December 7, 2022, via harmo, an electronic medication notebook smartphone app operated by harmo Co, Ltd (part 1 in Figures 1 and 2). The data set also included the PHR, such as the dispensing history of the respondent users and drug formulation information in the package insert.

The target population comprised 49,505 men and women aged 20 years or older who had been prescribed either tablets or capsules within 90 days from 2 weeks (November 16) before the survey distribution date (December 1). We sent notifications about the questionnaire to the target population, including a link to the survey details, through the harmo smartphone app. Users were then asked to provide consent and respond through the linked platform. The questionnaire included the following items: whether they found the tablets or capsules too large to swallow, the names of the medications that corresponded to the above question, and whether they had swallowing difficulties. Since larger numbers of questions are associated with lower response rates, it was necessary to set the lowest number of questions possible. Thus, we set simple questions to evaluate whether dysphagia was present without using objective measures such as the 10-item Eating Assessment Tool in this study [8].

For PHR, we obtained the following information from the data stored in the electronic medication notebook (part 2 in Figures 1 and 2) while maintaining anonymity: dispensing history and basic information (gender and age).

**Figure 3:**
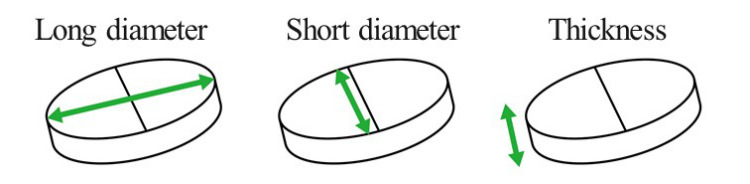
Schematic showing the measurement methods of long diameter, short diameter, and thickness (each unit is a millimeter).

For medication formulation information, we obtained the following information from the package inserts (part 3 in Figures 1 and 2): long diameter, short diameter, and thickness (Figure 3). At this time, we distributed tablets and capsules of the same formulation. This was because a previous study conducted by Kabeya et al [4] demonstrated that the difficulty of swallowing tablets and capsules is almost identical. The diameters were calculated as follows: diameter = long diameter = short diameter for regular circular formulations; capsule diameter = short diameter = thickness for capsule formulations; and diameter = long diameter = short diameter = thickness for spherical formulations.

### Outcomes

The primary outcome was the risk factors affecting the difficulty in swallowing tablets or capsules owing to their large size. The secondary outcomes were an appropriate index of the size of tablets or capsules difficult to swallow due to their large size, such as “long diameter + short diameter,” and a threshold for the size of tablets or capsules difficult to swallow due to their large size.

### Identification of Tablet or Capsule Size

We identified the names of medications in the dispensing history of those medications that were reported to be difficult to swallow owing to their large size in the questionnaire. Tablets or capsules with identifiable “long diameter,” “short diameter,” and “thickness” from the package insert were defined as “tablets or capsules difficult to swallow due to their large size” (parts 4 and 5 in Figure 1).

Tablets or capsules that could be identified from the dispensing history as being taken by the same patient or at the same period as tablets or capsules difficult to swallow due to their large size were defined as “concomitant tablets or capsules” and formed the control group (parts 6 and 7 in Figure 1). In Japan, tablets and capsules are sometimes powdered to make them easier to ingest, for example, by crushing the tablets or removing the powder from the capsule. In this study, we handled tablets and capsules as powders if instructions related to crushing could be obtained from the dispensing comments in the dispensing history.

### Search for Indices and Thresholds for the Size of Tablets or Capsules Difficult to Swallow

Based on the medication formulation information, 6 indices were set for each of the tablets or capsules that were difficult to swallow owing to their large size and concomitant tablets or capsules (used as controls; parts 8 and 9 in Figure 1), and the receiver operating characteristic (ROC) was calculated for each index. The index with the largest area under the receiver operating characteristic curve (AUC) was used as the best index of the size of tablets or capsules difficult to swallow because of their large size (part 10 in Figure 1).

From the generated ROCs, the point with the highest discriminative performance that maximized the Youden index was identified, and the optimal threshold for each index was calculated (part 11 in Figure 1) [9].

### Factor Analysis of Patients Who Tended to Find Swallowing Difficult Because of the Tablet or Capsule Size

Multivariate logistic regression analysis was performed to identify risk factors for patients who tended to find swallowing difficulties due to the size of the tablets or capsules. Based on information on patient preferences regarding tablets or capsules, the objective variable was defined as the presence or absence of tablets or capsules that were difficult to swallow because of their large size (part 4 in Figure 2). The following explanatory variables were set from patient preferences regarding tablets or capsules, PHR, and formulation information (part 5 in Figure 2): gender, age, dysphagia, number of oral medications per day (including powdered medicine and liquid), number of tablets or capsules per day, number of tablets or capsules per timing, total size of tablets or capsules per day, taking powdered medicine, taking liquid, single-packaging, and taking large tablets or capsules (Note S1 in Multimedia Appendix 1).

Among these explanatory variables, “number of oral medications per day (including powdered medicine and liquid),” “number of tablets or capsules per day,” “tablets or capsules per timing,” and “total size of tablets or capsules per day” were assumed to have correlations, and therefore were selected from the clinical perspective that the correlation ratio with “taking large tablets or capsules” was the lowest and could be easily calculated by health care workers. For variables that were not binary, cutoff values were determined based on the results of stratified group comparisons and were classified as binary (part 6 in Figure 2). In addition, sensitivity analysis was conducted to evaluate whether different factors were extracted when patients were stratified based on their age (by dividing the identified cutoff value). For this analysis, the same explanatory variables for formal analysis were used except for age. The significance level was set at 5%, and the odds ratios (ORs) and 95% CIs were calculated for each explanatory variable (part 7 in Figure 2).

To estimate the risk of a combination of multiple factors, a decision tree analysis was performed using the significant risk factors in the multivariate logistic regression analysis (part 8 in Figure 2). Chi-Squared Automatic Interaction Detection (CHAID), which divides features using a *χ*^2^ test, was also used [10]. CHAID is an algorithm that creates multiple cross-tabulation tables between the objective variable and each explanatory variable, and then branches the tree by selecting the variable that is most significant from the *χ*^2^ test. The analysis conditions were based on a previous study using the same method [11].

Python (version 3.8.8; Anaconda Inc) was used for data sampling, index search, threshold calculation, and multivariate logistic regression analysis, and SPSS Statistics (version 28.0.1.0; IBM Corp) was used for decision tree analysis.

### Ethical Consideration

This study was a self-administered, anonymous questionnaire survey (web-based questionnaire) and all data obtained in this study were anonymized and deidentified. Only those who gave their consent to the questionnaire survey and the provision of PHR owned by the harmo Co, Ltd were asked to complete the questionnaire. This study’s protocol was approved by the ethics committee of the Keio University Faculty of Pharmacy (221111-1). No incentives were distributed for survey respondents.

## Results

### Questionnaire Results on Tablet or Capsule Size

Out of 49,505 patients, 5528 (11.2%) patients opened the survey request notification, 1976 (4%) patients accessed the questionnaire page, and 1501 (3%) patients responded to the questionnaire. Among them, 212 (14.1%) patients answered, “There are tablets or capsules difficult to swallow due to their large size.” Regarding dysphagia, 189 (12.6%) patients reported that they “usually found it difficult to swallow while eating” (Table 1).

**Table 1:** Questionnaire results (N=1501).

Questions and answers			Patients, n (%)
**Are there any tablets or capsules difficult to swallow due to their large size?**			
	Yes	212 (14.1)	
	No	1271 (84.7)	
	Do not know	18 (1.2)	
**Do you usually find it difficult to swallow when eating?**			
	Yes	189 (12.6)	
	No	1283 (85.5)	
	Do not know	29 (1.9)	

### Search for Indices and Thresholds for the Size of Tablets or Capsules Difficult to Swallow

**Figure 4:**
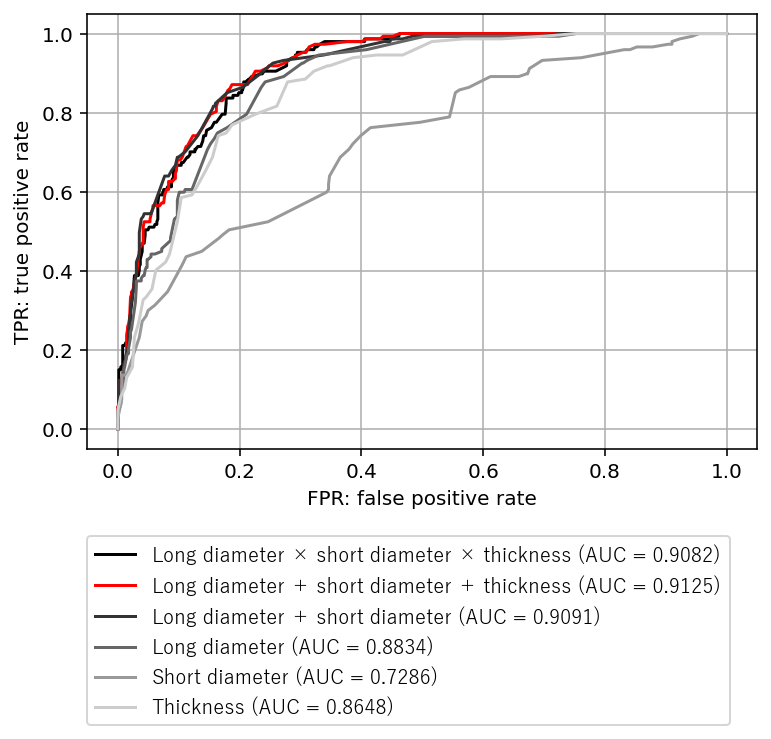
ROC and AUC of medical tablets or capsule size based on 6 indices. AUC: area under the receiver operating characteristic curve; FPR: false positive rate; ROC: receiver operating characteristic; TPR: true positive rate.

Figure 4 shows the ROC of 147 tablets or capsules that were difficult to swallow because of their large size and 624 control tablets or capsules and their AUCs. The highest AUC was obtained for “long diameter + short diameter + thickness” (0.9125). Table 2 presents the threshold values calculated from the illustrated ROCs. The threshold value was calculated to be 21.5 mm for “long diameter + short diameter + thickness,” which had the highest AUC in the index search.

**Table 2:** Calculation of the size threshold of tablets or capsules that patients find difficult to swallow due to their large size.

Index	Average (SD)	Threshold	Sensitivity	Specificity
Long diameter × short diameter × thickness	278.2 (208.4)	279.5	0.89115	0.78045
Long diameter + short diameter + thickness	20.0 (4.7)	21.5	0.87048	0.81250
Long diameter + short diameter	16.2 (3.7)	17.4	0.85034	0.82051
Long diameter	8.9 (3.1)	8.7	0.87755	0.75801
Short diameter	7.3 (1.5)	7.2	0.76190	0.58494
Thickness	3.8 (1.2)	4.0	0.87755	0.72115

### Factor Analysis of Patients Who Tended to Find Swallowing Difficult Because of the Tablet or Capsule Size

Of the 1501 patients, 86 patients with missing data were excluded. The analysis included 1415 patients (132 patients with tablets or capsules swallowing difficulties owing to the size of the formulations and 1283 patients without swallowing difficulties). As shown in Table S1 in Multimedia Appendix 1, “number of oral medications per day (including powdered medicine and liquid),” “number of tablets or capsules per day,” “number of tablets or capsules per timing,” and “total size of tablets or capsules per day” showed strong correlations. We selected “number of tablets or capsules per timing” as a variable because of its lowest correlation ratio with “taking large tablets or capsules” (Table S2 in Multimedia Appendix 1) and the clinical perspective that it can be easily calculated by health care workers.

The nonbinary variables “age” and “number of tablets or capsules per timing” were classified as binary variables by setting cutoff values (aged 50 years and number of tablets or capsules per timing: 4) based on the results of stratified group comparison (Tables S3 and S4 in Multimedia Appendix 1).

An elevated percentage of patients who showed difficulties swallowing tablets or capsules because of their size were more likely to be female or aged younger than 50 years (Table S5 in Multimedia Appendix 1). The results of the multivariate logistic regression analysis are presented in Table 3. The variables identified as risk factors were aged <50 years (OR 1.59, 95% CI 1.03-2.44), female (OR 2.54, 95% CI 1.70-3.78), dysphagia (OR 3.54, 95% CI 2.22-5.65), and taking large tablets or capsules (OR 9.74, 95% CI 5.19-18.29). Similar trends were obtained from the sensitivity analysis when patients were divided based on whether they were “younger than 50 years” or “aged 50 years or older” (Tables S6 and S7 in Multimedia Appendix 1).

**Table 3:** Multivariate logistic regression analysis (N=1415).

Variable name	Odds ratio (95% CI)	P value
Aged <50 years	1.59 (1.03-2.44)	.04
Female	2.54 (1.70-3.78)	<.001
Dysphagia	3.54 (2.22-5.65)	<.001
Number of tablets or capsules per timing ≥4^a^	1.12 (0.74-1.68)	.59
Taking powdered medicine	0.98 (0.62-1.53)	.91
Taking liquid	0.83 (0.22-3.12)	.79
Single-packaging	0.34 (0.04-2.98)	.33
Taking large tablets or capsules^b^	9.74 (5.19-18.29)	<.001

^a^The number of tablets or capsules per time indicates the number of tablets or capsules with the same dosing time (time to reach the maximum number of pieces) as tablets or capsules that were difficult to swallow because of their large size in patients who answered that they had tablets or capsules that they found large and difficult to swallow. In the case of patients who answered that they had no tablets or capsules that were difficult to swallow because of their size, the number was defined as the number of tablets or capsules at the same dosing time (time to reach the maximum number of pieces) as the latest prescription tablet or capsule.

^b^Taking large tablets or capsules indicates the presence or absence of tablets or capsules in the medications taken that exceeded the threshold for the index of tablets or capsule size (long diameter + short diameter + thickness was 21.5 mm).

**Figure 5:**
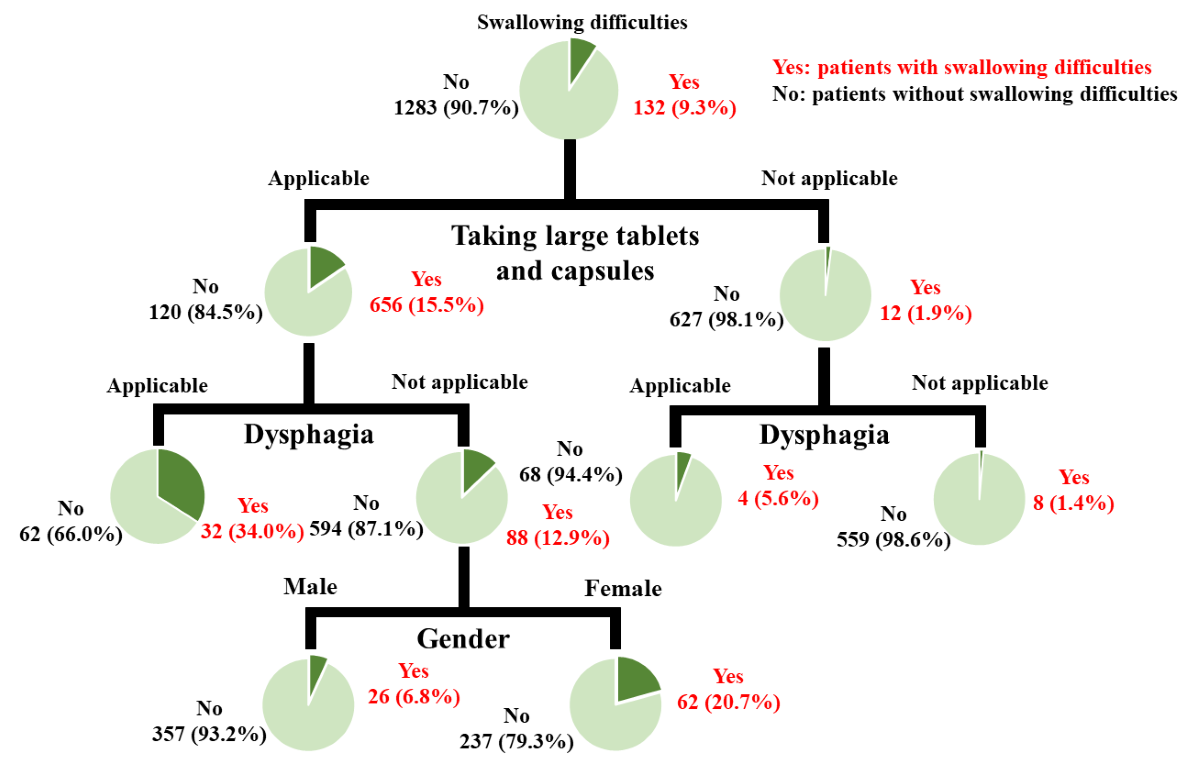
Decision tree analysis.

In the subsequent decision tree analysis based on these risk factors, the risk was higher in patients with “taking large tablets or capsules” and “dysphagia” (percentage of patients who reported difficulty in swallowing: 32 out of 94 patients, 34% patients). Additionally, even in the absence of dysphagia, the risk was higher in patients “taking large tablets or capsules” and who were “female” (percentage of patients who reported difficulty in swallowing: 62 out of 299 patients, 20.7% patients; Figure 5).

## Discussion

### Principal Results

In this study, we conducted an analysis using a questionnaire with an electronic medication notebook, PHR, and tablets or capsules formulation information.

We identified the size index and threshold of tablets or capsules that patients find difficult to swallow, as well as the risk factors for patients who find tablets or capsules “too large to swallow.” The threshold for the best index was “long diameter + short diameter + thickness” = 21.5 mm, and the risk factors were aged <50 years, female sex, dysphagia, and the use of large tablets or capsules. In addition, patients with “taking large tablets or capsules” and “having dysphagia” and those with “taking large tablets or capsules” and “no dysphagia” and “female” were at relatively high risk.

For the index search and threshold calculation, the PHR-based study design defined “difficult-to-swallow” as well as “not difficult-to-swallow” tablets or capsules. Compared with a previous study based on a geometric analysis of histograms [5], this study enabled conducting a threshold or indicator search using the ROC-AUC and obtaining more generalized findings while strengthening the validity of the previous report.

### Results of the Questionnaire About the Size of Tablets or Capsules

Although it is difficult to estimate the percentage of people who find tablets or capsules difficult to swallow because of the differences in methods and study populations, it has been shown that 10%-40% of adult patients in general practice find tablets or capsules difficult to swallow [12-15]. Among the patients evaluated in this study, 212 (14.1%) patients felt that some tablets or capsules were difficult to swallow because their sizes fell within the range of values obtained in these studies. A 2007 US survey on dysphagia in general practice patients aged 18 years showed a prevalence of dysphagia of 22.6% [16]. In this study, 189 (12.6%) patients reported that they usually found it difficult to swallow when eating showed a lower percentage than that in the previous report. This may be due to the lower response rate among older adults in this study (who generally have an elevated prevalence of dysphagia) and racial differences than those in previous studies.

### Indicator Search and Threshold Calculation for Tablets or Capsules Difficult to Swallow Owing to Their Large Size

The AUC (0.9125) for “long diameter + short diameter + thickness” was extremely good in the index search and threshold calculation, suggesting that this index has high discrimination power as an index of medical tablets or capsule size. Furthermore, the threshold value of 21.5 mm for “long diameter + short diameter + thickness” was similar to that in a previous report [4] (22 mm: a small clinical study of 40 patients) and was considered to have a certain validity.

### Multivariate Logistic Regression Analysis

In multivariate logistic regression analysis, aged <50 years, which was identified as a risk factor, was at odds with the general perception that dysphagia due to tablets or capsules was more frequent in the older age group. However, the result was consistent with those of previous studies, in which younger patients were more likely to report difficulty in swallowing tablets or capsules than older patients [12,17].

These results suggest that the measures commonly taken to make medication easier for older patients may not be considered for younger patients. It is also possible that this was due to younger and middle-aged patients’ unfamiliarity with taking tablets or capsules compared to older patients who take medications daily and are accustomed to taking tablets or capsules. However, we believe that further verification of whether age is a true risk factor for swallowing difficulties is needed. This is because age was not extracted in the DT model, that is, its association with swallowing difficulties may be relatively weaker than that of other variables. In addition, as described above, age was likely influenced by external factors such as the absence of measures to make it easier to swallow tablets and capsules.

Female gender has been identified as a risk factor in several studies [12,13,18]. Schiele et al [12] attributed this to physiological processes and anatomical differences in the dimensions and function of the mouth, pharynx, upper esophageal sphincter, and esophagus, as well as a higher incidence of depression and anxiety disorders. Dysphagia was identified as a risk factor in a previous study in which patients attending general practice were asked to complete a questionnaire, and the same trend was observed [12]. The same trend was observed for large tablets or capsules because the size of the solid dosage form has been cited as a cause of dysphagia in many studies [1-5]. Furthermore, the high OR (9.74) and 95% CI (5.19-18.29) suggest that the size of the medical tablet or capsule is the most important factor in avoiding risk among the risk factors identified in this study. The validity of the threshold value of 21.5 mm for “long diameter + short diameter + thickness,” which was calculated only for patients who reported having a difficult-to-swallow medical tablet or capsule, was strengthened by conducting a factor analysis that included patients who reported having no difficult-to-swallow tablets or capsules.

### Decision Tree Analysis

This study investigated the risk estimation of swallowing by combining factors in a decision tree analysis. In the CHAID model used in this study, the order of the branches reflects the importance of each feature. Since the explanatory variable in the highest branch was “taking large tablets or capsules,” it was the explanatory variable that contributed the most to the objective variable. The risk was higher in patients with “taking large tablets or capsules” and “dysphagia” (32/94, 34% of patients). Additionally, even in the absence of dysphagia, the risk was higher in patients “taking large tablets or capsules” and who were “female” (62/299, 20.7%). In contrast, the risk was low in patients who did not take large tablets or capsules (12/639, 1.9%). These results identified a more detailed group of patients for whom health care workers should provide easier-taking measures and advice when prescribing medications compared with previous studies.

### Clinical Application

Tablets or capsules that surpass the determined threshold should be subject to formulation adjustments, such as transformation into orally disintegrating tablets, divisible through a dividing line, or formulation of smaller dose units. It is important to design a medical tablet or capsule formulation that does not exceed the threshold size to reduce swallowing difficulty. Furthermore, it is important to design prescriptions and provide guidance to younger patients aged younger than 50 years to ease intake of such formulations, ultimately enhancing compliance with prescribed doses. Moreover, the implementation of straightforward monitoring practices, such as asking patients about their daily experiences with swallowing difficulties during meals and beverages, can serve as an effective means of identifying individuals at a higher risk of dysphagia. Early intervention, including a potential change in dosage form, can then be promptly initiated in response to these findings.

### Limitations

This study acknowledges limitations stemming from the characteristics of the electronic medication notebook and the questionnaire design. Among the limitations associated with the electronic medication notebook, population bias is a notable concern. Furthermore, in this questionnaire survey conducted using harmo, there was a tendency for younger to middle-aged people rather than older people, which could be attributed to the penetration rate. The skewed distribution contrasts with the age profile of hospital outpatients in Japan [19], raising the possibility that the results may have been influenced by this sampling bias. Furthermore, our focus was on those individuals among older people who can complete the questionnaire using their smartphones. A survey conducted among residents of a nursing home revealed that 68% of older participants experienced difficulty in swallowing at mealtime [20]. Therefore, the older people who find it difficult to swallow tablets or capsules may be underestimated. In addition, the medication intake in patients cannot be ensured in that patients actually take the medication, as recorded in the dispensing history, and there is no guarantee that all tablets or capsules taken by patients are recorded in the electronic medication notebook. Furthermore, there is no certainty that all prescribed medications are recorded in the electronic medication book as part of the dispensing history.

The limitations stemming from the questionnaire-based design include the potential for response bias, as this study relies on active participation and interest in swallowing tablets or capsules. Another factor was the high exclusion rate due to missing data, resulting in a reduced sample size from 212 to 132 patients in the factor analysis. Furthermore, we could not use objective measures of dysphagia (such as the 10-item Eating Assessment Tool) due to the need to minimize the number of included questions.

Despite these limitations, this study presents an innovative approach aimed at generating tablets or capsules and prescription considerations using the data collected from electronic medication notebooks. The novelty lies in risk estimation through the combination of various factors and the validation of the usefulness of the PHR infrastructure. We anticipate that the use of PHR will continue to grow in the future, and this study serves as a pioneering step in this promising direction.

### Conclusions

Combining the information on patient preferences regarding tablets or capsules obtained from the questionnaire survey of the electronic medication notebook, the user’s PHR and medication formulation information revealed that the most suitable indicator for determining the size of medical tablets or capsules causing swallowing difficulties is “long diameter + short diameter + thickness,” with a threshold of 21.5 mm. In addition, factors such as “aged <50 years,” “female,” “dysphagia,” and “taking large tablets or capsules” were identified as patient backgrounds and prescribing backgrounds that are likely to cause patients to feel difficulty in swallowing large tablets or capsules. Furthermore, patients who ingested “large tablets or capsules” and had “dysphagia” were shown to be at an elevated risk of difficulty in swallowing. Although some sampling biases could not be avoided due to the low response rate (1501/49,505, 3% of patients) and specific survey population (ie, only smartphone users), these findings have significant implications for optimizing the design of patient-friendly formulations and prescriptions.

## Appendix

Multimedia Appendix 1 A note and tables.

## Abbreviations

AUC: area under the receiver operating characteristic curve
CHAID: Chi-Squared Automatic Interaction Detection
EAT-10: 10-Item Eating Assessment Tool
OR: odds ratio
PHR: personal health record
ROC: receiver operating characteristic

## Data Availability

The questionnaires and PHR data for this study are available upon reasonable request.
