# Supplementary material for: Factor Analysis of Patients Who Find Tablets or Capsules Difficult to Swallow Due to Their Large Size: Using the Personal Health Record Infrastructure of Electronic Medication Notebooks
Source: J Med Internet Res. 2024 Apr 24;26:e54645. doi: 10.2196/54645 (PMC11079767; doi:10.2196/54645)
Supplement: Multimedia Appendix 1 [file jmir_v26i1e54645_app1.docx]

## **Note S1.** Definition of explanatory variables for logistic regression analysis.

For those who answered that they had tablets or capsules that they found difficult to swallow due to their size, “the medications taken” were defined as tablets or capsules that are difficult to swallow due to their large size, plus any other medication taken regularly at the same period. For those who answered that they had no tablets or capsules that they found difficult to swallow due to their size, “the medications taken” were defined as the latest prescription tablet or capsule they were taking regularly, plus any medications they were taking regularly at the same period.

The number of oral medications per day (including powdered medicine and liquid) was defined as the number of types of medications internally, including powdered medicines and liquids among “the medications taken.”

Number of tablets or capsules per day was defined as the number of types of tablets or capsules taken internally among “the medications taken.”

The number of tablets or capsules per time point was defined as the number of tablets or capsules with the same dosing time (time to reach the maximum number of pieces), for example, after breakfast, as tablets or capsules that were difficult to swallow because of their large size in the case of patients who answered that they had tablets or capsules that they found large and difficult to swallow. In the case of patients who answered that they had no tablets or capsules that were difficult to swallow because of their size, the number was defined as the number of tablets or capsules at the same dosing time (time to reach the maximum number of pieces) as the latest prescription tablet or capsule.

The total number of tablets per capsule per day was defined as the sum of the sizes of the tablets or capsules among the medications administered. The best index for the size of tablets or capsules in search for indexes is used for the size of the tablets or capsules.

Single packaging refers to a measure in which medications taken simultaneously are packaged together in a single bag to prevent mistakes or the loss of medications. This is common practice in Japan. The definition of single packaging was based on drug and dosage comments in the dispensing history of the medications taken.

Taking large tablets or capsules was defined as the presence or absence of tablets or capsules in medications that exceeded the threshold for the index of tablets or capsules size.

Orally disintegrating (OD) tablets are generally considered easy to swallow. However, since the majority of patients were taking multiple medications, we could not assess the impact of OD tablets on swallowing difficulties in this study.

**Table S1.** Correlation coefficient between covariates.

| Variable Name | Number of oral medications per day (including powdered medicine and liquid) | Number of tablets or capsules  per timing | Total size of tablets or capsules per day^d^ |
| --- | --- | --- | --- |
| Number of tablets or capsules per day^a^ | 0.98 | 0.85 | 0.97 |
| Number of oral medications per day (including powdered medicine and liquid)^b^ | ----- | 0.87 | 0.98 |
| Number of tablets or capsules per timing^c^ | ----- | ----- | 0.85 |

^a^The number of tablets or capsules per day indicates the number of types of tablets or capsules taken internally among the medications taken.

^b^The number of oral medications taken per day (including powdered medicine and liquids) indicates the number of types of medications taken internally, including powdered medicines and liquids.

^c^The number of tablets or capsules per timing indicates the number of tablets or capsules with the same dosing time (time to reach the maximum number of pieces) as tablets or capsules that were difficult to swallow because of their large size in patients who answered that they had tablets or capsules that they found large and difficult to swallow. In the case of patients who answered that they had no tablets or capsules that were difficult to swallow because of their size, the number was defined as the number of tablets or capsules at the same dosing time (time to reach the maximum number of pieces) as the latest prescription tablet or capsule.

^d^The total size of tablets or capsules per day indicates the sum of the sizes of the

tablets or capsules, among the medications administered. The best index for the

size of tablets or capsules in search for indexes is used for the size of the tablets or

capsules.

**Table S2.** Correlation ratios between “taking large tablets or capsules” and other covariates.

| Variable Name | Correlation　ratio |
| --- | --- |
| Number of oral medications per day (including powdered medicine and liquid)^a^ | 0.173 |
| Number of tablets or capsules per day^b^ | 0.184 |
| Number of tablets or capsules per timing^c^ | 0.170 |
| Total size of tablets or capsules per day^d^ | 0.256 |

^a^The number of tablets or capsules per day indicates the number of types of tablets or capsules taken internally among the medications taken.

^b^The number of oral medications taken per day (including powdered medicine and liquids) indicates the number of types of medications taken internally, including powdered medicines and liquids.

^c^The number of tablets or capsules per time indicates the number of tablets or capsules with the same dosing time (time to reach the maximum number of pieces) as tablets or capsules that were difficult to swallow because of their large size in patients who answered that they had tablets or capsules that they found large and difficult to swallow. In the case of patients who answered that they had no tablets or capsules that were difficult to swallow because of their size, the number was defined as the number of tablets or capsules at the same dosing time (time to reach the maximum number of pieces) as the latest prescription tablet or capsule.

^d^The total size of tablets or capsules per day indicates the sum of the sizes of the

tablets or capsules among the medications administered. The best index for the

size of tablets or capsules in search for indexes is used for the size of the tablets or

capsules.

**Table S3.** A stratified comparison of age between the patients with and without tablets or capsules that they found difficult to swallow due to their size (N=1415).

| Age | Patients with tablets or capsules that they found difficult to swallow due to their size (n = 132) | Patients without tablets or capsules that they found difficult to swallow due to their size (n = 1,283) |
| --- | --- | --- |
| 20s, n (%) | 4 (3.0) | 31 (2.4) |
| 30s, n (%) | 14 (10.6) | 57 (4.44) |
| 40s, n (%) | 23 (17.4) | 169 (13.2) |
| 50s, n (%) | 32 (24.2) | 340 (26.5) |
| 60s, n (%) | 33 (25.0) | 394 (30.7) |
| 70s, n (%) | 22 (16.7) | 248 (19.3) |
| 80s, n (%) | 3 (2.27) | 41 (3.20) |
| 90s, n (%) | 1 (0.8) | 3 (0.2) |

**Table S4.** A stratified comparison of “number of tablets or capsules per timing” between the patients with and without tablets or capsules that they found difficult to swallow due to their size (N=1415).

| Number of tablets or capsules per timing^a^ | Patients with tablets or capsules that they found difficult to swallow due to their size (n = 132) | Patients without tablets or capsules that they found difficult to swallow due to their size (n = 1,283) |
| --- | --- | --- |
| ≥0 and <1, n (%) | 0 (0.0) | 4 (0.3) |
| ≥1 and <2, n (%) | 17 (12.9) | 247 (19.3) |
| ≥2 and <3, n (%) | 19 (14.4) | 238 (18.6) |
| ≥3 and <4, n (%) | 16 (12.1) | 207 (16.1) |
| ≥4 and <5, n (%) | 23 (17.4) | 174 (13.6) |
| ≥5 and <6, n(%) | 14 (10.6) | 130 (10.1) |
| ≥6 and <7, n(%) | 13 (9.8) | 76 (5.9) |
| ≥7 and <8, n(%) | 9 (6.8) | 67 (5.2) |
| ≥8 and <9, n(%) | 4 (3.0) | 47 (3,7) |
| ≥9 and <10, n(%) | 4 (3.0) | 31 (2.4) |
| ≥10, n(%) | 13 (9.8) | 62 (4.8) |

^a^The number of tablets or capsules per timing indicates the number of tablets or capsules with the same dosing time (time to reach the maximum number of pieces) as tablets or capsules that were difficult to swallow because of their large size in patients who answered that they had tablets or capsules that they found large and difficult to swallow. In the case of patients who answered that they had no tablets or capsules that were difficult to swallow because of their size, the number was defined as the number of tablets or capsules at the same dosing time (time to reach the maximum number of pieces) as the latest prescription tablet or capsule.

### **Table S5.** A stratified comparison of explanatory variables between the patients with and without tablets or capsules that they found difficult to swallow due to their size (N=1415).

| Variable Name | Patients with tablets or capsules that they found difficult to swallow due to their size (n = 132) | Patients without tablets or capsules that they found difficult to swallow due to their size (n = 1,283) |
| --- | --- | --- |
| *Age* |  |  |
| <50, n (%) | 41 (31.1) | 257 (20.0) |
| >50, n (%) | 91 (68.9) | 1,026 (80.0) |
| *Female*, n (%) | 86 (65.2) | 521 (40.6) |
| *Dysphagia*, n (%) | 32 (24.2) | 130 (10.1) |
| *Number of tablets or capsules per timing^a^* |  |  |
| <4, n (%) | 37 (28.0) | 524 (40.8) |
| ≥4, n (%) | 54 (40.9) | 502 (39.1) |
| *Taking powdered medicine* | 35 (26.5) | 263 (20.5) |
| *Taking liquid* | 3 (2.3) | 25 (1.9) |
| *Single-packaging* | 1 (0.8) | 11 (0.9) |
| *Taking large tablets or capsules^b^* | 120 (90.9) | 656 (51.1) |

^a^The number of tablets or capsules per timing indicates the number of tablets or capsules with the same dosing time (time to reach the maximum number of pieces) as tablets or capsules that were difficult to swallow because of their large size in patients who answered that they had tablets or capsules that they found large and difficult to swallow. In the case of patients who answered that they had no tablets or capsules that were difficult to swallow because of their size, the number was defined as the number of tablets or capsules at the same dosing time (time to reach the maximum number of pieces) as the latest prescription tablet or capsule.

^b^Taking large tablets or capsules indicated the presence or absence of tablets or capsules in the medications that exceeded the threshold for the index of tablets or capsules size.

**Table S6.** Multivariate logistic regression analysis in patients younger than 50 years (N=298).

| Variable Name | Odds ratio (95% confidence interval) | *P*-value |
| --- | --- | --- |
| Female | 1.81 (0.83–3.93) | .13 |
| Dysphagia | 2.94 (1.22–7.10) | <.01 |
| Number of tablets or capsules per timing ≥4^a^ | 1.78 (0.84–3.76) | .13 |
| Taking powdered medicine | 0.87 (0.37–2.02) | .74 |
| Taking liquid | 2.01 (0.15–26.11) | .59 |
| Single-packaging | 0.00 (Not Applicable) | .99 |
| Taking large tablets or capsules^b^ | 11.34 (3.27–39.32) | <.01 |

^a^The number of tablets or capsules per timing indicates the number of tablets or capsules with the same dosing time (time to reach the maximum number of pieces) as tablets or capsules that were difficult to swallow because of their large size (in patients who answered that they had tablets or capsules that they found large and difficult to swallow). In the case of patients who answered that they had no tablets or capsules that were difficult to swallow because of their size, the number was defined as the number of tablets or capsules at the same dosing time (time to reach the maximum number of pieces) as the latest prescription tablet or capsule.

^b^Taking large tablets or capsules indicated the presence or absence of tablets or capsules in the medications that exceeded the threshold for the index of tablets or capsules size.”

**Table S7.** Multivariate logistic regression analysis in patients aged 50 years or older (N=1117).

| Variable Name | Odds ratio (95% confidence interval) | *P*-value |
| --- | --- | --- |
| Female | 2.82 (1.77–4.49) | <.01 |
| Dysphagia | 3.73 (2.15–6.47) | <.01 |
| Number of tablets or capsules per timing ≥4^a^ | 0.91 (0.56–1.49) | .72 |
| Taking powdered medicine | 1.10 (0.64–1.88) | .73 |
| Taking liquid | 0.65 (0.13–3.2) | .60 |
| Single-packaging | 0.35 (0.04-3.09) | .34 |
| Taking large tablets or capsules^b^ | 9.25 (4.45–19.24) | <.01 |

^a^The number of tablets or capsules per timing indicates the number of tablets or capsules with the same dosing time (time to reach the maximum number of pieces) as tablets or capsules that were difficult to swallow because of their large size (in patients who answered that they had tablets or capsules that they found large and difficult to swallow). In the case of patients who answered that they had no tablets or capsules that were difficult to swallow because of their size, the number was defined as the number of tablets or capsules at the same dosing time (time to reach the maximum number of pieces) as the latest prescription tablet or capsule.

^b^Taking large tablets or capsules indicated the presence or absence of tablets or capsules in the medications that exceeded the threshold for the index of tablets or capsules size.”
